# Supplementary material for: A UMLS-based spell checker for natural language processing in vaccine safety
Source: BMC Med Inform Decis Mak. 2007 Feb 12;7:3. doi: 10.1186/1472-6947-7-3 (PMC1805499; doi:10.1186/1472-6947-7-3)
Supplement: Additional file 13 — RAP application directory. Sets up directory for RAP (RDF application for PHP) source code [file 1472-6947-7-3-S13.gz › rap/doc/phpdoc/dataset/IteratorAllGraphsMem.html]

xml version="1.0" encoding="iso-8859-1"?


Docs For Class IteratorAllGraphsMem


## Class IteratorAllGraphsMem

Description

Description |
Methods (details)

Implementation of a Graph iterator.

This Iterator should be used in a for-loop like: for($iterator = $dataset->listGraphs(); $iterator->valid(); $iterator->next()) { $currentResource=$it->current(); };

History: <LI>05-03-2005 : First version of this class.</LI>

- access: public
- author: Daniel Westphal <at d-westphal dot de mail at d-westphal dot de>
- version: V0.9.1

Located in /dataset/IteratorAllGraphsMem.php (line 30)

Method Summary

Description |
Methods (details)

IteratorAllGraphsMem
IteratorAllGraphsMem
(dataset &$namedGraphSet)

mixed
&current
()

integer
key
()

void
next
()

void
rewind
()

boolean
valid
()

Methods

Description |
Methods (details)

Constructor IteratorAllGraphsMem (line 68)

Constructor.

- access: public

IteratorAllGraphsMem

IteratorAllGraphsMem
(dataset &$namedGraphSet)

- dataset
  &$namedGraphSet

current (line 113)

Returns the current item

- access: public

mixed

&current
()

key (line 124)

Returns the key of the current item

- access: public

integer

key
()

next (line 101)

Moves Iterator to the next item in the list

- access: public

void

next
()

rewind (line 79)

Resets iterator list to start

- access: public

void

rewind
()

valid (line 91)

Says if there are additional items left in the list

- access: public

boolean

valid
()

Documentation generated on Thu, 7 Jul 2005 13:41:46 +0200 by phpDocumentor 1.3.0RC3
